# A comparative examination of the health status of earthquake-affected and non-earthquake-affected adolescents in Yushu

**DOI:** 10.3389/fpubh.2022.976075

**Published:** 2022-10-21

**Authors:** Fangjie Zhao, Bihan Tang, Hongyang Yang, Jing Wu, Qi Chen, Lulu Zhang, Xu Liu

**Affiliations:** ^1^Department of Health Service, Second Military Medical University, Shanghai, China; ^2^Xinhua Hospital, School of Medicine, Shanghai Jiao Tong University, Shanghai, China; ^3^School of Nursing, Second Military Medical University, Shanghai, China; ^4^Department of Health Statistic, Second Military Medical University, Shanghai, China

**Keywords:** health services, adolescent, earthquakes, children, health status

## Abstract

**Background:**

Yushu, Qinghai Province, which is located in the remote Tibetan Plateau in western China, was struck by a disastrous earthquake in 2010.

**Methods:**

This study aimed to compare the health status of adolescents who had (Exp-Group) and had not (Non-Group) experienced the Yushu earthquake, 7 years after it occurred; additionally, group-specific predictors of health status were identified. A cross-sectional study was adopted among students from two junior schools in Yushu, whereby two groups were compared. Descriptive statistics, *t*-tests, Wilcoxon rank-sum tests, Kruskal-Wallis H tests, and stepwise linear regression were used to analyze data.

**Results:**

Exp-Group scored higher than Non-Group on Physiological Component Summary (PCS) but not on Mental Component Summary (MCS). Among Exp-Group participants, lower PCS scores were predicted for “house damaged,” “injured,” “family member injured,” and “family member or friend dead.” Lower MCS scores were predicted by “family member or friend dead.” Among Non-Group participants, PCS scores were predicted by “residence” and “family member or friend dead.” Lower MCS scores were predicted by “not living with parents.”

**Conclusion:**

Lower PCS and MCS scores of Exp-Group adolescents mainly contributed to earthquake-related injuries, while lower PCS and MCS scores of Non-Group are related to poor living conditions and the fact of the left-behind child.

## Background

On 14 April 2010, the city of Yushu, in Qinghai Province, which is a remote plateau in western China, was struck by an earthquake measuring 7.1 on the Richter scale (maximum intensity: 8), which resulted in 2,698 deaths, 12,135 injuries, and an average elevation of 4,493 m. It has been evaluated that experiencing an earthquake has an impact on both the physical and mental health of survivors (1–5), but most of them have evaluated the impact of disasters 1 or 2 years after the disaster, in terms of mortality, morbidity, post-traumatic stress disorder (PTSD), and health-related quality of life (HRQoL) (6–10). Few studies have examined the long-term health status of child and adolescent survivors in the years following an earthquake (11–13). To the best of our knowledge, there has been no study that has compared the health status of children and adolescents who have and have not experienced an earthquake. We were interested in examining whether this earthquake had a long-term impact on the physical and mental health of children and adolescents 7 years after the event.

Geographically, the city of Yushu in Qinghai Province is a remote plateau in western China, and high morbidity and mortality and poor health status among nomadic herdsmen who live in pastures are global public health problems (11, 14–17). In fact, this is an important aspect of the broader health inequalities that exist worldwide (14, 15). Compared with Southeast China, socioeconomic and infrastructure development in this region is relatively limited (11). Therefore, many parents choose to work in the city and leave their children with their grandparents or other relatives; this has led to an increase in the number of “left-behind children” (18). Given this unique arrangement, the health status of children and adolescents living in the Qinghai Plateau is worthy of attention. Many researchers have focused on the health status of the residents of the plateau; however, the samples used in these studies have primarily consisted of local adults (14–16). Consequently, there have only been a few studies that have examined the health status of children and adolescents in remote areas of western China. These studies have found that children who reside in this area tend to be in poor health; this may be attributable to factors such as high altitude, lack of nutrition-related knowledge, and unhealthy eating habits (19, 20).

Additionally, cultural difference is another important dimension that is worthy of consideration because it has significant implications for health (21). Furthermore, since there are many ethnic minorities in Yushu, there are nuanced cultural differences that could influence the health status of children and adolescents. For example, it has been found that maternal and child health (MCH) outcomes and service coverage are poorer among ethnic minorities than among the Han population in western China (21).

The aim of this study was to examine the long-term effects of the Yushu earthquake on the physical and mental health of children and adolescents residing in remote Tibetan plateaus in western China. This was to be achieved by comparing the physical and mental health status of children and adolescents who had and had not experienced the Yushu earthquake. The resultant findings were to be used as an empirical base on which recommendations for long-term health interventions for children and adolescents who have experienced earthquakes can be found. The Chinese version of the Short Form-12 (SF-12), a simplified version of the 36 Schedule Health Surveys (SF-36), was used, which has been used in numerous studies worldwide, including items that have been drawn from scales measuring bodily pain, general health, vitality, and social functioning; additionally, it contains two items from each of the scales measuring physical functioning, role-physical, role-emotional, and mental health (22).

## Methods

### Patient and public involvement

A cross-sectional survey was conducted in August 2017, at which time 7 years had passed since the Yushu earthquake (23, 24). In China, most primary schools have 6 grades (i.e., grades 1–6), which are equivalent to grades 1–6 in the Western education system; the ages of students who belong to these grades typically range from 7 to 13 years. The local summer vacation in Yushu, which spans from May to June, is 2 months ahead of the summer vacation in most regions of China; therefore, we conducted our research study in August. We randomly selected 2 out of 25 primary schools for participation in our research study: Qironglian College Primary School in Maozhuang Township, Nangqian County, and Second Boarding Primary School in Baizha Town, Nangqian County.

To examine the impact of the Yushu earthquake on adolescent health, we divided the research participants into two groups, namely, those who had experienced the earthquake (Exp-Group) and those who had not experienced the earthquake (Non-Group). Group-specific multiple linear regressions were conducted to identify the factors that influence adolescent health in each group.

The number of students in Qironglian College Primary School is 433, and the number of students in the Second Boarding Primary School is 245. Owing to missing responses, four questionnaires were excluded (3 lacked the data on ‘experienced Yushu earthquake or not' and 1 refused to participate in the questionnaire). Therefore, the total number of participants in this study was 674.

### Data collection

The survey was conducted by eight undergraduates from the Navy Military Medical University. The project leader conducted a centralized training in Shanghai for the eight investigators before the initiation of the research, whereby he briefed them about the details of the study and answered their questions about the study. The investigation was conducted between 10 and 24 August 2017. The survey was conducted within the confines of school classrooms. The researchers briefed the respondents about the details of the questionnaire and answered relevant questions that helped them complete the questionnaire.

### Measures

The social and demographic information that was collected in this study pertained to the following: “age,” “family living area,” and “living with parents.” Students who did not live with their parents were referred to as left-behind children, which is defined as those who live with their parents for <30 days in 1 year.

Information related to students' experiences of an earthquake was elicited using questions adapted from an earthquake exposure scale. The items included “residence,” “living with parents,” “experienced Yushu earthquake,” “house damaged in earthquake,” “injured in earthquake,” “family member injured in earthquake,” and “family member or friend dead in earthquake” (25). The resultant questionnaire included the following items: “experienced the 2010 Yushu earthquake,” “house damaged in earthquake,” “injured in earthquake,” “family member injured in earthquake,” and “family member or friend dead in earthquake” (25).

Student health status was assessed using the Chinese version of the Short Form-12 (SF-12), which is a simplified version of the 36 Schedule Health Surveys (SF-36). The Chinese version of the SF-12 has been validated using samples of Chinese youth (26). Specifically, participants were asked to respond to the SF-12 on the basis of their personal experiences in the past 4 weeks. The SF-12, which has been used in numerous studies worldwide, includes items that have been drawn from scales measuring bodily pain, general health, vitality, and social functioning; additionally, it contains two items from each of the scales measuring physical functioning, role-physical, role-emotional, and mental health (22). The SF-12 yields the following two summary scores that serve as indicators of overall physical and mental functioning: Physiological Component Summary (PCS) score and Mental Component Summary (MCS) score (27). In this study, the PCS and MCS scores were calculated using the method proposed by Ware et al. (28); the higher the score, the better the health status. Only 2–3 min is required to complete SF-12 (4).

### Data analysis

All analyses were conducted using the Statistical Package for the Social Sciences SPSS 21.0 (IBM, Armonk, NY, USA). Descriptive statistics (i.e., frequencies, percentages, means, and standard deviations) were calculated. Subsequently, nonparametric analyses were used (i.e., Wilcoxon rank-sum tests for two-group comparisons and the Kruskal-Wallis H tests for multiple-group comparisons). Multiple linear regression analysis was used to identify the group-specific factors that predict PCS and MCS scores (29). Meanwhile, a stepwise linear regression analysis was also conducted; the thresholds for a variable to be entered into the analysis and retained in the model were specified as 0.05 and 0.10, respectively. The resultant beta coefficients (B), standardized error of the coefficient (SEB), and other relevant statistics were used in the interpretation of the results. The PCS and MCS scores of the Exp-Group and Non-Group were, respectively, compared with those of the general adolescent population in China using the one-sample *t*-test (26). Additionally, scores on the PCS and MCS were compared across the Exp-Group and the Non-Group using the independent-sample *t*-test. A *p*-value that was < 0.05 was considered to be indicative of a statistically significant result.

## Results

### Demographics and earthquake-related experiences

Participants' ages at the time of the survey ranged from 8 to 15 years. Of the 674 participants, 590 (87.5%) lived on pasture lands and 624 (92.4%) lived with their parents. Over half of the sample (346; 51.3%) had experienced the Yushu earthquake in 2010. Furthermore, with regard to the consequences of the earthquake, 41 (6.1%) participants reported that their family members had been injured; 120 (17.8%) reported that family members or friends had died; 53 (15.4%) reported that their houses had been damaged; and 13 (3.8%) reported that they had sustained injuries (Appendix 1).

The mean PCS score and MCS score of all 674 participants are 49.66 ± 7.94 and 40.99 ± 8.27, respectively. The mean PCS score of the Exp-Group (48.76 ± 7.87) is significantly lower than that of the Non-Group (50.64 ± 7.89) (*p* < 0.05). However, the mean MCS score of the Exp-Group (40.56 ± 8.56) is not statistically different from that of the Non-Group (41.42 ± 7.91) (*p* = 0.18) (Appendix 2).

The mean PCS score of the Exp-Group (48.76 ± 7.84) is significantly lower than that of the general adolescent population in China (49.8 ± 6.6) (26) (*p* = 0.02). The mean MCS score of the Exp-Group (40.56 ± 8.56) is also lower than that of the general adolescent population in China (45.4 ± 9.7) (26) (*p* < 0.05) (Appendix 2).

The mean PCS score of the Non-Group (50.65 ± 7.89) is not significantly different from that of the general adolescent population in China (49.8 ± 6.6) (26) (*p* = 0.05). In contrast, the mean MCS score of the Non-Group (41.42 ± SD 7.91) is significantly lower than that of the general adolescent population in China (45.4 ± 9.7) (26) (*p* < 0.05) (Appendix 2).

### Factor-wise differences in PCS and MCS within the exp-group

The results presented in Table 1 suggest that the PCS scores obtained by the Exp-Group significantly differed across groups subsumed by the following variables: residence, house damaged in earthquake, injured in earthquake, family member injured in earthquake, and family member or friend dead in earthquake. In contrast, statistical differences in MCS scores emerged only across groups subsumed by one factor, i.e., family member or friend dead in earthquake.

**Table 1:** Nonparametric rank-sum test of differences in PCS and MCS scores across the exp-group and non-group.

**Variable**	**N**	**%**	**Exp-group: PCS**		**Exp-group: MCS**		***N***	**%**	**Non-group: PCS**		**Non-group: MCS**	
			**Median (IQR)**	***P* (95%CI)**	**Median (IQR)**	***P* (95%CI)**			**Median (IQR)**	***P* (95%CI)**	**Median (IQR)**	***P* (95%CI)**
Total	346		49.16 (11.61)		40.63 (12.71)		328		52.61 (11.69)		42.57 (11.70)	
Residence				0.027 (0.023, 0.029)		0.84 (0.834, 0.848)				0 (0, 0)		0.713 (0.710, 0.728)
Downtown	9	2.60	45.63 (11.36)		37.36 (18.08)		5	1.50	57.84 (12.86)		47.34 (12.49)	
Country	31	9.00	46.17 (11.07)		40.77 (13.87)		39	11.90	43.97 (13.42)		41.28 (10.77)	
Pasture	306	88.40	49.69 (11.44)		40.63 (12.34)		284	86.60	53.11 (11.03)		42.58 (11.35)	
Living with parents				0.488 (0.478, 0.498)		0.112 (0.107, 0.119)				0.056 (0.052, 0.061)		0.001 (0, 0.001)
Yes	320	92.50	49.09 (11.76)		40.73 (12.41)		305	93.00	52.84 (11.71)		42.87 (10.71)	
No	26	7.50	50.88 (14.78)		36.77 (13.41)		23	7.00	47.52 (8.48)		33.76 (11.93)	
House damaged in earthquake				0 (0, 0)		0.328 (0.317, 0.335)				—		—
Yes	53	15.40	44.83 (17.79)		40.68 (9.63)		—	—	—		—	
No	293	84.60	50.22 (11.51)		40.54 (12.90)		—	—	—		—	
Injured in earthquake				0 (0, 0)		0.15 (0.210, 0.226)				—		—
Yes	13	3.80	41.14 (9.97)		43.68 (13.32)		—	—	—		—	
No	333	96.20	49.65 (11.33)		40.54 (12.48)		—	—	—		—	
Family member injured in earthquake				0.005 (0.003, 0.006)		0.080 (0.075, 0.086)				0.584 (0.579, 0.599)		0.172 (0.170, 0.185)
Yes	30	8.70	44.53 (11.63)		44.03 (12.73)		11	3.40	54.95 (16.19)		38.47 (16.67)	
No	316	91.30	49.61 (11.35)		40.29 (12.57)		317	96.60	52.61 (11.68)		42.61 (11.49)	
Family member or friend dead in earthquake				0 (0, 0)		0.019 (0.018, 0.023)				0.003 (0.001, 0.004)		0.631 (0.626, 0.645)
Yes	94	27.20	44.75 (11.31)		41.14 (12.17)		26	7.90	44.11 (12.00)		39.37 (18.42)	
No	252	72.80	50.59 (10.87)		40.15 (12.86)		302	92.10	52.83 (11.07)		42.63 (11.11)	

Exp-Group, Group of participants who had experienced the Yushu earthquake; Non-Group, Group of participants who had not experienced the Yushu earthquake; Gen-Group, General adolescent population in China.

The results of the multiple stepwise regression analyses (Table 2) revealed that PCS scores were significantly predicted by the following variables: house damaged in earthquake, injured in earthquake, family member injured in earthquake, and family member or friend dead in earthquake. MCS scores were significantly predicted by only one variable, i.e., family member or friend dead in earthquake (Table 3).

**Table 2:** Results of the multiple stepwise regression analysis for variables predicting PCS scores across the exp-group and non-group.

**Predictor**	**Exp-group: Initial model**		**Exp-group: REGRESSION model**		**Non-group: initial model**		**Non-group: REGRESSION model**	
	***β***	***P***	***β***	***P***	***β***	***P***	***β***	***P***
Residence (Downtown)								
Country	0.01	0.94			−0.52	0.00	−0.50	0.00
Pasture	0.11	0.28			−0.22	0.14		
Living with parents (Yes: 1, No: 2)	0.03	0.63			−0.07	0.20		
House damaged in earthquake (Yes: 1, No: 2)	0.14	0.01	0.15	0.01	—	—		
Injured in earthquake (Yes: 1, No: 2)	0.11	0.05	0.13	0.02	—	—		
Family member injured in earthquake (Yes: 1, No: 2)	0.11	0.04	0.12	0.02	0.01	0.93		
Family member or friend dead in earthquake (Yes: 1, No: 2)	0.20	0.00	0.21	0.00	0.18	0.00	0.19	0

Exp-Group = Group of participants who had experienced the Yushu earthquake; Non-Group = Group of participants who had not experienced the Yushu earthquake; Gen-Group = General adolescent population in China.

**Table 3:** Multiple stepwise regression analysis for variables predicting MCS scores across the exp-group and non-group.

**Predictor**	**Exp-group: Initial model**		**Exp-group: REGRESSION model**		**Non-group: Initial model**		**Non-group: REGRESSION model**	
	***β***	***P***	***β***	***P***	***β***	***P***	***β***	***P***
Residence (Downtown)								
Country	0.00	0.97			−0.18	0.25		
Pasture	0.08	0.50			−0.16	0.29		
Living with parents (Yes: 1, No: 2)	−0.09	0.09			−0.20	0	−0.20	0.00
House damaged in earthquake (Yes: 1, No: 2)	−0.01	0.81			—	—		
Injured in earthquake (Yes: 1, No: 2)	−0.01	0.81			—	—		
Family member injured in earthquake (Yes: 1, No: 2)	−0.09	0.10			0.08	0.13		
Family member or friend dead in earthquake (Yes: 1, No: 2)	−0.13	0.02	−0.14	0.01	0.04	0.50		

Exp-Group, Group of participants who had experienced the Yushu earthquake; Non-Group, Group of participants who had not experienced the Yushu earthquake; Gen-Group, General adolescent population in China.

### Factor-wise differences in PCS and MCS within the non-group

As presented in Table 1, PCS scores obtained by the Non-Group significantly differed across subgroups that were differentiated on the basis of the following factors: residence and family member or friend dead in earthquake. MCS scores significantly differed across groups subsumed by one variable, i.e., living with parents.

Multiple stepwise regression analysis (Table 2) revealed that PCS scores were significantly predicted by the following two variables: residence and family member or friend dead in earthquake. In contrast, MCS scores were significantly predicted by only one variable, i.e., living with parents (Table 3).

## Discussion

Experiencing an earthquake can have long-term physiological and psychological effects on adolescents (11, 12, 30). This study aimed to compare the health status of children and adolescents who had and had not experienced the Yushu earthquake, 7 years after it occurred, and identify group-specific predictors of physical and mental health (26). The difference in PCS scores between groups might be attributable to the participants' experiences of the Yushu earthquake and the relatively backward living conditions in this region (16). In contrast, the difference in MCS scores between groups might be attributable to the experiences that the Yushu earthquake entailed and the lack of long-term psychological intervention following the disaster.

Among participants who belonged to the Exp-Group, PCS scores were found to vary according to houses damaged as a result of the earthquake or not and sustain injuries or not. These results are inconsistent with past findings that these variables have no significant effect on the PCS scores of survivors of natural disasters (31). However, in other studies, poor housing conditions, such as living in temporary shelters and lacking clean water and food, were found to have a negative impact on physical health 1–18 months after the disaster (4, 32, 33). Our study found that experiencing an earthquake has a long-term influence on PCS. For example, the collapse of one's house and the consequent injuries that lead to physical disabilities and movement disorders may directly reduce the PCS scores of survivors. Overall, the results suggest that it is important to develop long-term follow-up interventions that address earthquake-related injuries and promote the recovery of survivors.

Among participants of the Exp-Group, the death of a family member or friend in the earthquake was found to be negatively related to physical and mental health. This finding, which concurs with those of past studies, has been found to be more likely to occur among people who have poor mental health at the time of occurrence of a natural disaster (2, 12, 34). Other studies have shown that children who are extremely fearful in the face of a disaster are more likely to have poor mental health and are more prone to post-traumatic stress disorder, depression, and anxiety (35–37). Since our study findings show that earthquakes have an impact on adolescent psychology even after 7 years, appropriate psychological counseling is needed to reduce its potentially adverse psychological effects. Furthermore, long-term psychological interventions must be established in order to improve the psychological health of survivors in earthquake-affected areas.

Among participants of the Non-Group, the place of residence was found to have significant implications for physical health. Many studies have found that rural and urban living places differentially influence children's nutritional intake and eating habits, which in turn have an impact on their PCS scores (16, 38–40). This finding may be attributable to the polarizing gap between the rich and the poor that has resulted from the rapid urbanization of China.

Among participants of the Non-Group, those who reported living with their parents obtained higher MCS scores than those who did not. This might be the case because China has been rapidly urbanizing in the past few decades. In fact, one out of ten individuals are immigrants who are seeking employment opportunities in both rural and urban areas. Accordingly, quite a few participants reported that they did not live with their parents.

Evidence from national studies suggests that a stable family environment contributes to the healthy development of children (16, 38, 41, 42). In addition, left-behind children have been found to demonstrate negative and unhealthy behavior patterns, negative mood, and poor psychosocial health (18, 43–45). Additionally, since the Yushu area is a plateau with a scattered population, the students had to be sampled from boarding schools. Given that they had just started school, their inability to adapt to the school atmosphere and its living conditions might have contributed to low MCS scores.

## Limitations

This study has a few limitations that merit further discussion. First, this study adopted a cross-sectional research design, whereby those who had and had not experienced an earthquake were compared. Second, although this study was conducted by well-trained researchers, data collection relied on self-reports of child survivors 7 years after the Yushu earthquake; these self-reports might have been adversely affected by recall bias. Despite these limitations, this study is the first to analyze the health status of children and adolescents who had experienced the Yushu earthquake. It is also one of the few cross-sectional studies that have focused on long-term health status and associated risk factors among children and adolescents who had experienced earthquakes in the remote Tibetan plateau of western China. Further study will focus on the effect of psychological interventions on the long-term health status of children and adolescents who had experienced earthquakes.

## Conclusion

This study found that the lower PCS and MCS scores of children and adolescents who had experienced the Yushu earthquake are mainly contributed to earthquake-related injuries, while the lower PCS and MCS scores of those who had not experienced the Yushu earthquake are related to poor living conditions and the fact of being a left-behind child. In conclusion, these children and adolescents who have experienced the Yushu earthquake must be provided with specialized care and long-term psychological interventions; such services must also be rendered to those who were injured in the earthquake and those who have lost family members or friends as a result of the earthquake. In contrast, children and adolescents who have not experienced the Yushu earthquake, especially left-behind children, can be helped by improving their school atmosphere and living conditions.

## Data availability statement

The original contributions presented in the study are included in the article/Supplementary material, further inquiries can be directed to the corresponding author/s.

## Author contributions

XL, BT, and FZ discussed and developed the question for this study. XL and HY abstracted data from medical records. QC and JW carried out all analysis. BT and FZ wrote the first draft of this paper, which was reviewed LZ and XL. XL is the guarantor. All authors have read and approved the manuscript, involved in the interpretation and discussion of results, and agreed on the final draft of this study.

## Funding

Work on this manuscript was sponsored by the National Natural Science Foundation of China XL, Grant Number [71774166]; BT, Grant Number [71804186]; LZ, Grant Number [72174204, 91224005]; JW, Grant Number [71904200], Chinese National Defence Research Program of Science and Technology XL, Grant Number [2019-JCJQ-JJ-065], San Hang Program of Naval Medical University XL and BT, and Shanghai Pujiang Program BT, Grant Number [18PJC116], Military Key Disciplines Construction Project (LZ, Grant 03). The design and data collection of the study were funded by the funding granted 71774166, 71804186, 71904200, and 72174204. The analysis, and interpretation of data were funded by the funding granted 91224005, 2019-JCJQ-JJ-065, and 18PJC116. The writing of the manuscript was funded by the funding granted 03 and San Hang Program of Naval Medical University XL and BT.

## Conflict of interest

The authors declare that the research was conducted in the absence of any commercial or financial relationships that could be construed as a potential conflict of interest.

## Publisher's note

All claims expressed in this article are solely those of the authors and do not necessarily represent those of their affiliated organizations, or those of the publisher, the editors and the reviewers. Any product that may be evaluated in this article, or claim that may be made by its manufacturer, is not guaranteed or endorsed by the publisher.

## Abbreviations

PCS: Physiological Component Summary
MCS: Mental Component Summary.
