# Supplementary material for: A comparative examination of the health status of earthquake-affected and non-earthquake-affected adolescents in Yushu
Source: Front Public Health. 2022 Oct 21;10:976075. doi: 10.3389/fpubh.2022.976075 (PMC9645053; doi:10.3389/fpubh.2022.976075)
Supplement: Supplementary file 1 [file Data_Sheet_1.docx]

Appendix 1. Demographics and earthquake-related experiences of the study participants

| Variables | N/n | | Percentage (%) |
| --- | --- | --- | --- |
|  |  |  |  |
| Total | 674 |  | |
| Residence |  |  | |
| Downtown | 14 | 2.1 | |
| Country | 70 | 10.4 | |
| Pasture | 590 | 87.5 | |
| Living with parents |  |  | |
| Yes | 623 | 92.4 | |
| No | 51 | 7.6 | |
| Experienced Yushu earthquake |  |  | |
| Yes | 346 | 51.3 | |
| No | 328 | 48.7 | |
| House damaged in earthquake |  |  | |
| Yes | 53 | 7.9 | |
| No | 621 | 92.1 | |
| Injured in earthquake |  |  | |
| Yes | 13 | 1.9 | |
| No | 661 | 98.1 | |
| Family member injured in earthquake |  |  | |
| Yes | 41 | 6.1 | |
| No | 633 | 93.9 | |
| Family member or friend dead in earthquake |  |  | |
| Yes | 120 | 17.8 | |
| No | 554 | 82.2 | |

Appendix 2. Significance of the difference in mean PCS and MCS scores across the Exp-Group, Non-Group, and Gen-Group

| Items | Group comparisons | t | 95% CI |
| --- | --- | --- | --- |
| Physical component summary (PCS) score | Exp-Group vs. Non-Group | -3.10 | -3.03, -0.65 |
|  | Non-Group vs. Gen-Group | 1.84 | -0.56, 1.65 |
|  | Exp-Group vs. Gen-Group | -2.46 | -1.87, -0.21 |
| Mental component summary (MCS) score | Exp-Group vs. Non-Group | -1.35 | -2.15, 0.35 |
|  | Non-Group vs. Gen-Group | -9.00 | -4.80, -3.08 |
|  | Exp-Group vs. Gen-Group | -10.52 | -5.74, -3.93 |

*Note.* Exp-Group = Group of participants who had experienced the Yushu earthquake; Non-Group = Group of participants who had not experienced the Yushu earthquake; Gen-Group = General adolescent population in China.
